# The Impact of Physical Activity at School on the Nutritional Behavior of Overweight Children

**DOI:** 10.3390/nu17243905

**Published:** 2025-12-13

**Authors:** Katarzyna Ługowska, Bożena Baczewska, Joanna Trafiałek, Wojciech Kolanowski

**Affiliations:** 1 Faculty of Medical and Health Sciences, University of Siedlce, 08-110 Siedlce, Poland; katarzyna.lugowska.zdoz@uws.edu.pl; 2 Faculty of Health Sciences, Medical University of Lublin, 20-400 Lublin, Poland; 3 Institute of Human Nutrition Sciences, Warsaw University of Life Sciences–SGGW, 02-776 Warsaw, Poland; 4 Healthcare Institute, State University of Applied Sciences in Przemyśl, 37-700 Przemyśl, Poland

**Keywords:** children obesity, BMI, fat mass, nutritional behavior, physical activity

## Abstract

**Background/Objectives:** Low physical activity (PA) is becoming an increasingly serious health problem among overweight school-age children. This study aimed to evaluate the influence of elevated PA during school hours on the nutritional behavior and fat mass of overweight and obese children. **Methods:** The study involved 11-year-old children (*n* = 148) who were overweight and obese. In the control group, children received physical education lessons in the standard dimension (4 h a week) while the intervention group received 10 h. Body mass index (BMI), fat mass (FM), and nutritional behavior were analyzed. **Results:** Compared to baseline, at the end of the intervention, the proportion of obese children increased in the control group and decreased in the intervention group. Regarding nutritional behavior, low consumption of vegetables, fruits, whole grains, poultry, and fish was observed. After a 12-month period, the intervention group showed a slight decrease in the consumption of fruits, vegetables, cold cuts, fried dishes, sweets, and fast food, and an increase in the consumption of white bread, whole-grain bread, poultry, red meat, and dairy products (milk, yogurt). In the control group, children exhibited an average increase in the consumption of fruits, vegetables, poultry, red meat, sweets, and fast food **Conclusions:** Although extended physical activity during school hours was correlated with a decrease in the number of obese children, the observational nature of the study precludes the drawing of definitive conclusions. The intervention may have contributed to an increase in energy expenditure, which could account for the improvements in BMI and FM. Nevertheless, the impact on nutritional behavior was limited.

## 1. Introduction

Overweight and obesity in childhood are among the most serious public health problems. Many studies confirm that early implementation of healthy nutritional behavior and increasing physical activity (PA) levels in the school setting can contribute to reducing excess body weight and improving dietary quality [1,2,3,4,5,6,7,8]. Systematically increasing the time spent on PA, combined with changes in dietary behaviors, leads to a lower body mass index (BMI), a reduction in body fat mass (FM), and beneficial changes in children’s diets [1,2,3]. A steady increase in the prevalence of obesity, a decline in physical activity levels, and a high consumption of low-nutrient foods are observed worldwide [1,4,5,6,7,8,9]. Currently, one in four children worldwide is overweight [1,4]. In Poland, obesity already affects approximately 20% of the school-age population, and another 20% are overweight [5,6,7]. Forecasts from the World Obesity Federation indicate that by 2030, obesity will affect approximately 254 million children and adolescents aged 5–19 [9]. Excess body weight significantly increases the risk of developing type 2 diabetes, hypertension, cardiovascular disease, and musculoskeletal disorders [10,11].

The main causes of overweight in childhood include an imbalance between energy intake and expenditure. One of the main causes of overweight in childhood is the imbalance between energy intake and energy expenditure [4,9,10]. Across the globe, there is an increase in the consumption of highly processed foods high in simple sugars and fats and low in vitamins and minerals [1,10,11,12]. At the same time, children’s lifestyles are becoming increasingly sedentary, leading to a systematic decline in physical activity [2]. According to the World Health Organization (WHO), children and adolescents should engage in moderate to vigorous physical activity, primarily aerobic, for at least 60 min daily [2]. Regular PA contributes to improved muscle mass and strength, beneficial changes in body composition, and a reduction in body weight and body fat in overweight or obese children [13,14].

Despite numerous well-documented benefits, over 80% of adolescents aged 11–17 do not engage in sufficient physical activity, and overweight children spend even more time sedentary [14,15]. Therefore, effective obesity treatment depends largely on reducing sedentary behavior and increasing motivation by fostering enjoyment of physical activity [16].

Numerous epidemiological studies confirm the effectiveness of the Mediterranean diet in preventing non-communicable diseases and maintaining a healthy body weight, especially when combined with regular physical activity [17,18,19]. For this reason, the Mediterranean diet is considered the most appropriate nutritional model for children [17,18,19]. In many countries, dietary recommendations are presented in the form of a “healthy eating plate”, which emphasizes the importance of consuming vegetables and fruits, whole grains, fish, low-fat dairy products, and eggs, while limiting the consumption of red meat, fried foods, and pastry products [20,21]. Water should be your main drink every day [20,21,22]. Lifestyle analyses indicated that overweight children are more likely to consume sweets, sweetened beverages, and high-calorie snacks. They snack more frequently between meals and consume regular meals less frequently, which helps maintain a positive energy balance [21,22].

Research published by Sunda et al. (2022) [23] indicated that PA levels are closely linked not only to physical fitness but also to children’s overall health, including body weight, nutrition, and health-promoting habits. These results highlight the importance of extracurricular activities and additional physical activity as potential support for overweight and obesity prevention in school populations. These aspects are strongly correlated with children’s level of understanding, education, and theoretical preparation for understanding the risks associated with physical inactivity and the negative consequences that result from it, including obesity and overweight [23].

Čaušević et al. (2023) [24] conducted research that demonstrated that children and adolescents who engaged in sports on a regular basis experienced natural changes in body composition as a result of variations in the rate of biological maturation. The authors underlined that body proportions, muscle mass, body fat mass, and general physical fitness can all be impacted by maturity level [24]. Children’s changes in body composition can be caused by both natural developmental processes and physical activity, as well as by involvement in educational and fitness programs [24].

Increased PA, especially when combined with nutritional education, can contribute to improving diet quality and developing healthier nutritional behavior [25,26,27,28]. The ISCOLE study results confirm the relationship between PA levels and nutritional behaviors, indicating that children who met PA recommendations were more likely to show healthier nutritional behavior [29]. Similar findings were reported by Kanellopoulou et al. (2021), who observed that participation in sports activities promoted improved nutritional behavior [30].

However, other studies have shown that physical activity alone does not always lead to lasting improvements in nutritional behavior [31]. The most effective programs are those that integrate both elements—promotion of PA and nutritional education—leading to an improvement in the quality of the diet and the level of activity [31].

Research verified that overweight children’s BMI and body composition can be positively influenced by increased PA during school hours [14,15,16]. However, there is a dearth of comprehensive analyses that compare the changes in nutritional behavior of overweight and obese children who are enrolled in supplemental PA programs. This study aimed to evaluate the influence of elevated physical activity (PA) during school hours on the nutritional behavior and fat mass of overweight and obese children over the course of a 12-month intervention. We hypothesized that overweight children would exhibit more favorable nutritional behavior following a 12-month PA intervention in comparison to the group that did not receive supplemental PA.

## 2. Materials and Methods

### 2.1. Participants

The research was done between 2018 and 2019. The study did not involve individual randomization. This was an observational cohort study that included 148 children born in 2007 who were overweight and obese. Children attended six elementary schools in Poland. The children involved in the study were from similar backgrounds and had comparable socioeconomic statuses. All the children lived in the same city. Most of the parents were well-educated, with over half of both mothers and fathers having higher education, nearly 43% having secondary education, and the remainder having vocational qualifications. Over 95% of the parents described their financial situation as average, approximately 4% as very good (above average), and less than 1% as poor (below average).

Children were divided into two groups. In the control group (SPA—standard PA, *n* = 88) children received physical education lessons (PE) in the standard dimension (4 h a week) while the intervention group (EPA—elevated PA, *n* = 60) children received 10 h. Participants were not randomly assigned to groups. Allocation resulted directly from class enrollment: children attending classes with an extended PE in the school curriculum formed the EPA group, while those enrolled in standard PE classes formed the SPA group. PE included fitness activities and team games. It wasn’t about coaching specific sports in depth; instead, the goal was to get more PA during school hours. The elevated PA program consisted of additional 45 min lessons delivered by certified PE teachers and included general fitness exercises, strengthening and coordination activities, and team games performed at moderate intensity. The content followed the school curriculum and was not additionally standardized or externally monitored.

The sample size was justified by power analysis in G*power software version 3.1.9.7 [32]. Power analysis performed before the study began showed that the minimum sample size required to detect a medium effect (d = 0.5) with α = 0.05 and a power of 0.80 was 128 participants (64 in each group). After applying inclusion and exclusion criteria, the number of participants decreased to 148, and ultimately, 112 children completed the study (43 in the intervention group and 69 in the control group). The high dropout rate resulted in a sample size in one of the groups that was lower than required for power analysis, which may have reduced the study’s final statistical power. To assess the potential impact of this phenomenon, an attrition analysis was performed, which revealed no significant differences in baseline characteristics between participants who completed the study and those who dropped out. Baseline characteristics (age, gender, BMI, and FM) were compared between children who completed the study and those who dropped out before its completion.

Before the study commenced, the children were apprised of the study’s objective and the confidentiality of the results. Written informed consent was obtained from parents or legal guardians. Additionally, organizational approval to conduct the study during school hours was obtained from the school principals and teachers. Consent was obtained from the principal and teachers. The project was approved by the Ethics Committee of the University of Siedlce.

Overweight and obesity were classified according to body mass index (BMI) values, calculated based on participants’ height and weight [33,34,35,36]. BMI was interpreted using national percentile charts developed for the Polish pediatric population, appropriate for age and gender [33,34,35,36].

Schools were selected using purposive sampling. All primary schools in Siedlce that offered both a standard physical education program and an enhanced physical activity program were invited to participate. As a result, six schools participating in the study implemented a standard PA program (SPA) and an extended PA program (EPA). Neither schools nor participants were randomized. Attendance at PE lessons was not objectively monitored in this part of the project. Data on extracurricular PA and fitness test performance were collected but will be analyzed and presented in a separate manuscript focusing on physical fitness tests.

Inclusion criteria considered children born in 2007, attending participating schools, and classified as overweight or obese based on national BMI percentiles. Exclusion criteria, defined a priori, included chronic diseases that may affect body weight or metabolism, medications that affect appetite or body weight, medical contraindications to physical activity, significant dietary restrictions (e.g., severe food allergies relevant to the questionnaire), and lack of parental consent. Participants who were absent during the measurement session were not excluded but were classified as lost to follow-up (dropped out). No exclusion criteria related to sex, previous sports participation, or psychological status were applied, as the study was designed to reflect a naturalistic school population of children with overweight or obesity. Participants were not stratified by sex. Medical examinations were not conducted as part of the protocol; however, parents were asked to report chronic illnesses or conditions that could affect participation in physical activity.

Participants who were absent during the final measurement session were not excluded based on criteria but were classified as lost to follow-up (dropout). These cases demonstrate attrition rather than exclusion.

**Figure 1:**
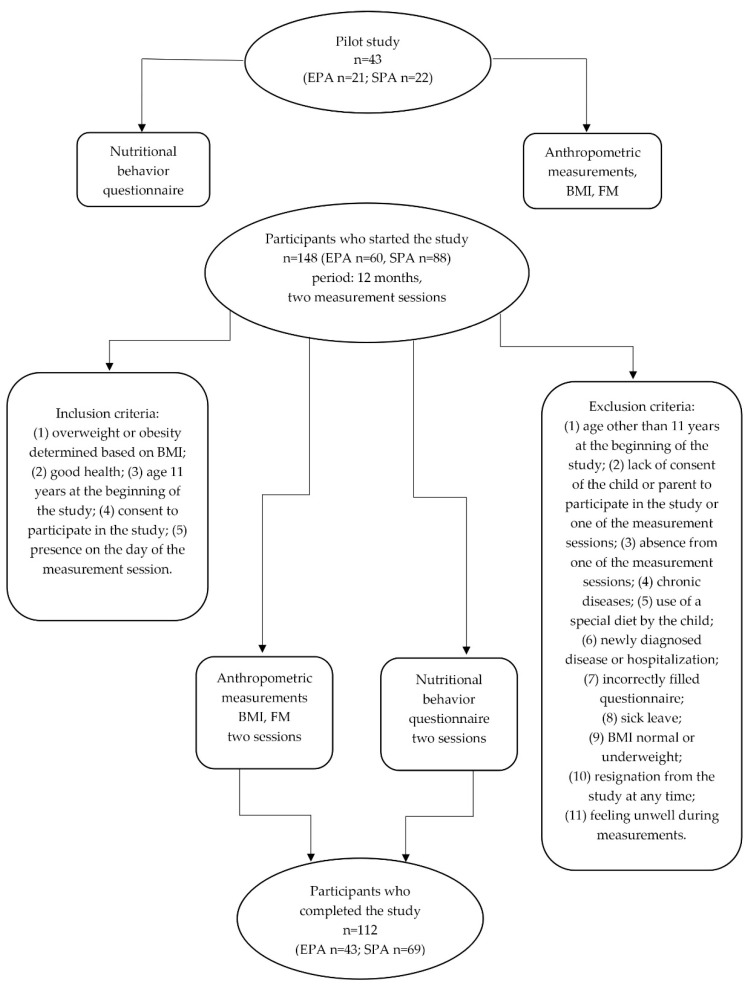
Flow chart of the study design and participants’ recruitment. EPA—elevated PA group; SPA—standard PA group; BMI—body mass index; FM—fat mass.

Details of the inclusion and exclusion criteria, as well as the participant flow through the study, are presented in the flowchart (Figure 1).

The methodological framework of the study is summarized in Figure 1, which illustrates the recruitment process, inclusion and exclusion criteria, group allocation, and participant retention.

### 2.2. Procedure

A qualified and trained team of dietitians took the measurements. There were two sessions of measurements: the first one was in May and June 2018, and the last one was in May and June 2019. Although the school year in Poland lasts 10 months, the measurements were planned for May–June 2018 and May–June 2019 to cover the full calendar year and simultaneously conduct the assessment under the same seasonal conditions, minimizing the impact of children’s summer activities. Measurements were taken with the same tools that were used in all schools. The BMI values and FM results were interpreted in accordance with age- and gender-appropriate percentile tables [34,35,36,37]. The measurements were taken using the same method we described in our earlier papers [38]. The present study is part of a broader research project previously described in several publications; however, unlike the earlier analyses, which included children across all BMI categories and often covered longer observation periods, the current paper focuses exclusively on children with overweight and obesity and provides a separate 12-month analysis conducted between 2018 and 2019 [38].

All measurements were conducted in the gym in the morning, in the presence of a teacher, in accordance with the study guidelines [34,35,36,37,38,39]. An anonymous questionnaire was implemented to assess dietary behavior. The results were compared with dietary recommendations [20,21]. Children completed the same questionnaire at the beginning and the end of the study.

According to the protocol, children were advised to eat a light meal on the day of the measurement and the day before the study. The research team advised that the most recent organized PA, except for necessities such as household chores, should be completed at least 12 h before the measurement. The children were instructed to refrain from consuming any food or beverages until the measurement was completed [40].

A coding system based on the BMI classification was used to determine the relationship between body mass index (BMI), body fat percentage (FM), and dietary behavior. Children with a normal weight were coded “2,” overweight “3,” and obese “4.”

**Figure 2:**
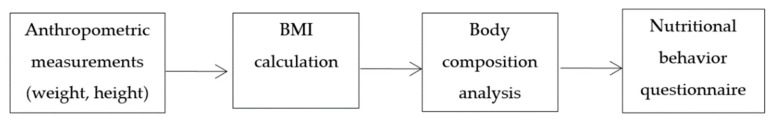
The sequence of measurements during each session. BMI—body mass index.

The study was divided into 4 steps (Figure 2). During the study session, height and weight measurements were taken, from which BMI was calculated. Each child then received a QEB questionnaire with the appropriate BMI code, allowing for anonymized analysis of the results—without revealing participants’ personal information. The data was analyzed only at the group level, any individual linkage was not possible, The questionnaires were completed individually in the assistance of the research team member, who provided clarifications as needed. After completing the questionnaire, a bioimpedance body composition analysis (BIA) was performed. The sequence of measurements during each session is illustrated in Figure 2.

The QEB questionnaires were fully anonymous and did not contain any personal data or information that could identify the participants. BMI and fat mass values were coded using identification numbers that likewise did not permit identification of individuals. These codes served solely to match measurement results with the corresponding questionnaires in a technical manner. All data were analyzed exclusively at the group level.

### 2.3. BMI Calculation

Body weight was measured to the nearest 0.1 kg using a medically approved bioelectrical impedance analyzer (Tanita SC-240MA, Tanita Corporation, Tokyo, Japan) [40,41,42,43]. Both devices were calibrated according to the manufacturer’s specifications before the start of each measurement session. Body height and weight were measured using standardized procedures. Height was assessed to the nearest 0.1 cm with a portable stadiometer Seca 214 equipped with an additional stabilizing accessory [41,42].

Each measurement (height and weight) was taken twice, and the mean value was used for further analysis. If the two measurements differed by more than 0.5 cm (height) or 0.2 kg (weight), a third measurement was performed and the median value was recorded.

BMI was calculated as weight (kg) divided by height squared (m^2^) and interpreted using national BMI percentile charts for the Polish pediatric population [34,35,36]. According to these reference values, BMI ≥ 85th percentile indicated overweight and BMI ≥ 95th percentile indicated obesity [34,35,36,37,38,39,40,41,42,43,44,45,46].

All measurements were performed in a standing position, without shoes, in light clothing, and following the recommended anthropometric protocol.

### 2.4. Fat Mass

The tests were always performed in a standing position in accordance with the measurement guidelines [42,43]. The Tanita SC-240 MA body composition analyzer, based on the bioelectric impedance technique, was employed for the estimation of FM [43]. The results that were gathered were only indicative due to the Tanita SC-240 MA analyzer’s failure to provide parameters for impedance, phase angle, reactance, and resistance. The values were related to the percentile charts [43,44], the detailed procedure is described in our previous works [38].

### 2.5. Nutritional Behavior

The analysis of dietary patterns was assessed using an anonymous survey consisting of 23 questions. The questions were based on the Eating Behavior Questionnaire (QEB) of the Polish Academy of Sciences [39]. Dietary behaviors were assessed using a reduced version of the KomPAN (QEB) questionnaire developed by the Polish Academy of Sciences. The version applied in this study was based on the original 2014 publication, which was the official version available at the time of study design. From the full questionnaire, 23 items were selected, corresponding to the food frequency section and key dietary habits relevant to overweight and obesity assessment in children. The selection followed the protocol of the main research project and focused on items essential for identifying changes in nutritional behavior.

Updated versions of the questionnaire published after 2018 (e.g., 2020 and 2024 revisions) were not used in this study. The QEB questionnaire, derived from the KomPAN tool developed by the Polish Academy of Sciences, was selected because it has been widely used in dietary behavior research among children and adolescents in Poland. The reduced 23-item version applied in this study focuses on core dietary habits and is linguistically and cognitively appropriate for 11-year-old respondents. Its structure minimizes respondent burden and has been previously applied in earlier publications within the same research project, demonstrating satisfactory usability in this age group.

The questionnaire contained closed questions with one possible answer to choose from. The questionnaires were completed anonymously and voluntarily. Unlike the gold standard of 24 h recall of food and beverage consumption over the previous 24 h, in this study, the authors assessed the frequency of food and drink consumption over a standard time frame, reflecting a typical diet.

Children were not time-limited. The study was preceded by instructions on how to understand and complete the questions properly. Questions were posed regarding the frequency of consumption of fish, eggs, red meat and poultry, cold cuts and sausages, milk and dairy products, white (wheat) and whole-grain bread, pasta, groats, butter, and lard. Additionally, inquiries were made regarding the frequency of vegetable, fruit, and fluid consumption. Final questions asked about the frequency of consumption of fast food, sweets, and salty snacks. Nutritional behaviors were referred to the recommendations and standards [20,21]. A detailed description can be found in our previous work [38]. Data supporting the results of this study are available in the Supplementary Materials accompanying this article. Additional tables and detailed statistical analyses are provided in the documents available for download.

Food and beverage consumption among children was analyzed based on mean values, considering consumption frequency. For products that constituted a significant part of the children’s daily diet (such as milk, yogurt, fatty cheeses, white bread, poultry, cold cuts, fried foods, fruit, vegetables, butter, and sweets), the combined percentage of responses “several times a week” and “daily” was calculated. For products consumed less frequently (including cottage cheese, whole-grain bread, eggs, fish, fast food, fruit juices, and carbonated drinks), the percentage of responses “once a week” was considered. This approach allowed for a differentiated presentation of data, tailored to the type of product and its typical consumption frequency in the pediatric population.

The questionnaire was developed based on the Eating Behavior Questionnaire (QEB) of the Polish Academy of Sciences, which has been previously used in studies of Polish school-aged children. In the present study, the internal consistency of the adapted tool was assessed. The content validity of the questionnaire was evaluated by experts in nutrition and public health, who confirmed the adequacy and comprehensibility of the items for the target age group. A pilot test conducted among 43 children verified that the questions were understandable and the response scale was appropriate.

Although the reduced version of the QEB questionnaire used in this study has not been formally validated in children with overweight and obesity, its structure is based on the original KomPAN tool developed by the Polish Academy of Sciences, which is well-established in dietary behavior research. The selected items represent core dietary habits relevant for this population, and the limitations associated with self-report and partial validation have been acknowledged in the manuscript.

However, it should be noted that the QEB questionnaire, in the form used in this study, was not formally validated in this specific population, which might limit the precision of the obtained results. In addition, the questionnaire was based on self-reported data and frequency of consumption rather than quantitative assessment, which may introduce reporting bias. This is discussed in the limitations section.

### 2.6. Statistical Analysis

Statistical calculations were performed using Microsoft Excel 365 (Microsoft, Corp., Washington, DC, USA) and Statistica 13 (Stat Soft, Krakow, Poland). The level of statistical significance was set at α ≤ 0.05. Student’s *t*-test, the Shapiro-Wilk test, the Mann-Whitney U test, and the effect size (ES) for the mean for children’s FM were calculated based on Cohen’s d. Effect sizes according to Cohen’s d were >0.2, >0.5, >0.8, and >1.3 for small, medium, large, and considerable effect sizes, respectively. In our work, we used an Effect size (d) of 0.8 with a power of 0.80. This meant that 112 participants would constitute a sufficient sample.

Analysis of covariance, paired *t*-test and *p*-value were used to assess the differences between the initial and final measurements and for average PA values. Furthermore, the confidence interval was determined at a 95% confidence level. The wider the interval, the greater the uncertainty about the exact location of the estimated parameter in the entire population.

The χ^2^ test was used to analyze nutritional behavior. The magnitude of the association between class profile and eating behaviors was evaluated using Cramer’s V (VC), which is derived from Pearson’s χ^2^ statistic [45]. When analyzing dietary behavior, we assumed average consumption of products and beverages among EPA and SPA. For frequently consumed products, such as milk, yogurt, fatty cheeses, white bread, poultry, cold cuts, fried dishes, fruits, vegetables, butter, and sweets, we combined the values from the multiple times a week and daily consumption indications. For less frequently consumed products, such as cottage cheese, whole-grain bread, fish, white rice and pasta, eggs, fish, fast food, fruit juices, and carbonated drinks, we used the data from the once-a-week consumption indication. Fisher’s exact test was applied to compare the frequencies of consumption of individual product groups between girls and boys as well as between eating behaviors in grades 4 and 5.

To reduce the variables, Principal Component Analysis (PCA) was performed. The PCA was prepared using the averaged results for BMI, FM, nutrition behavior, and PA level (SPA or EPA). PCA employed beneficial dietary behaviors (e.g., the consumption of fish, eggs, lean meat and cold cuts, whole grains, groats, vegetables, and fruits on a weekly basis) for both SPA and EPA children.

The aim of PCA was to find common factors, the so-called principal components, among BMI, FM, nutritional behavior, and PA level. The coefficients were in the range of 1; −1. The above methods allowed to determine the main factors influencing the results.

The analysis was conducted according to Per Protocol (PP) principles, including participants who completed all study phases and adhered to the study guidelines. This study used a per-protocol approach. Only participants who completed both measurement sessions were included in the final analyses. Missing values resulting from absence at the follow-up assessment were not imputed; instead, these participants were classified as lost to follow-up. No statistical methods, such as mean substitution or multiple imputations, were applied. To assess the potential impact of missing data, an attrition analysis comparing baseline characteristics between completes and dropouts was conducted. Due to attrition and the observational nature of the study, an intention-to-treat (ITT) analysis was not fully conducted. However, its limitations and the potential impact of data loss are discussed in detail in the limitations section. Additionally, attrition analysis was conducted using Student’s *t*-test to assess differences between participants who completed the study and those who dropped out. This analysis allowed us to assess anthropometric indicators and the potential impact of attrition on the results.

## 3. Results

### 3.1. Sample Characteristics

The children were an average age of 10.90 years at the beginning of the study and 11.90 years at the end. The study involved 148 children. However, the final analysis included results from 112 children (63% girls and 37% boys) who participated in both measurement sessions (EPA *n* = 43; girls 27; boys 16, and SPA *n* = 69; girls 43; boys 26) as a result of exclusions. The primary cause of exclusion was nonattendance during a measurement session or refusal to take part in the assessments.

There were no significant differences in height, weight, body fat, or BMI between participants who completed the study and those who dropped out in either the SPA or EPA groups (*p* > 0.05 for all comparisons) (Table 1).

In addition to comparing mean anthropometric parameters, we also examined the distribution of BMI categories (overweight vs. obesity) between participants who completed the study and those who dropped out. No significant differences were observed in either group, indicating that the dropout did not alter the proportion of overweight or obese children.

Over 44% of the kids in the study were obese (SPA 41.77%; EPA 44.77%), and about 56% were overweight (SPA 58.22%; EPA 57.22%). Trends in the variations in BMI categories between the baseline and the final measurements are shown in Table 2.

After one year of follow-up, both groups’ structures had changed. The percentage of children who were obese in the EPA group decreased from 44.77% to 37.87%, while children who were overweight increased from 57.22% to 62.15%.

### 3.2. Anthropometric Measurements

#### Anthropometric Characteristics of Children at Baseline and Follow-Up

Table 3 presents the initial and final values for the entire study group and individual subgroups (SPA, EPA). During the study period, children’s mean height increased by 6.18 cm (EPA: 5.46 cm; SPA: 6.91 cm; *p* = 0.124). Mean body weight increased by 6.95 kg across the cohort (SPA: 7.56 kg; EPA: 6.34 kg; *p* = 0.091).

FM percentage differed between groups, with lower values observed in EPA participants. In the SPA group, FM increased from 30.62% to 31.44% (*p* = 0.065), whereas in the EPA group it decreased from 28.08% to 26.98% (*p* = 0.071). In relative terms, FM decreased by 1.45% in EPA boys but increased by ~1% in SPA boys. Detailed values across measurement sessions are presented in Table 3.

### 3.3. Nutritional Bahavior

#### 3.3.1. Baseline

The supplementary materials include detailed data on the mean frequency of food, dish, and beverage consumption at baseline and at the end of the study, presented separately by class group and sex. In addition, the supplementary files contain graphical representations illustrating the average food and beverage intake among girls and boys, as well as among children in the EPA and SPA groups (see Supplementary Tables S1 and S2 and Figures S1 and S2).

At baseline, children in both groups most frequently consumed 4–5 meals per day, with similar patterns of lunch consumption (EPA 79.14%; SPA 82.90%; *p* = 0.061). Regular consumption of lunch was reported by 72% of children in the SPA group and 65.44% in the EPA group (*p* = 0.061). Approximately 18% of children in both groups consumed lunch only occasionally, while approximately 10% received money to purchase a meal. When it came to snacking between main meals, children in EPA and SPA groups most frequently chose fruit (42.58% and 40.90%, respectively; *p* = 0.210); sweet snacks (28.71% vs. 25.65%; *p* = 0.097) and salty snacks (19.32% vs. 22.93%; *p* = 0.081). No snacking was reported by only 10.51% of children in the SPA group and 9.08% of children in the EPA group between main meals (*p* = 0.091).

Regarding dairy products, SPA children more often consumed milk and cottage cheese, whereas EPA children reported higher intake of fatty cheeses. Butter was frequently present in the diets of both groups. It was daily consumed by 60.45% of children in the EPA group and 59.73% of children in the SPA group (*p* = 0.995). White bread was the most commonly consumed bakery product in both groups, while whole-grain bread was chosen more frequently by EPA children (*p* = 0.034). SPA children preferred white rice and pasta (*p* = 0.001), whereas EPA children more often selected whole-grain products (*p* = 0.014) (see Supplementary Table S1).

Poultry consumption was slightly higher in the EPA group, but red meat, cold cuts, eggs, and fish consumption did not differ significantly. Fish consumption was low. Children usually reported consuming fish once a week (EPA 63.81%; SPA 53.49%; *p* = 0.063) and several times a month (EPA 17.86%; SPA 17.92%; *p* = 0.107). A high consumption of fried meat and dough dishes was observed (see Supplementary Table S1).

Daily vegetable intake was more frequent in EPA children (*p* = 0.009), whereas SPA children consumed fruit more often. Legume intake was low in both groups. In the SPA group, 53.50% of children declared they never ate legumes, and the EPA group, 47% of children did not eat legumes (see Supplementary Table S1).

Sweets were consumed daily by approximately half of the children, with no significant between-group difference. Fast-food intake was also similar.

The most frequently chosen beverage in both groups was water (SPA 43.95%; EPA 53.58%; *p* = 0.601). Sugar-free tea consumption was reported by 22.98% of SPA children and 17.78% EPA children, while fruit juice consumption was reported by 10.46% and 15.17%, respectively. EPA children more frequently consumed sweetened beverages than SPA children (*p* = 0.028) (see Supplementary Table S1).

Most children in both groups believed that proper nutrition improves athletic performance (EPA 90.76%; SPA 94.39%; *p* = 0.047).

On average, at the beginning of the study, SPA children were characterized by more frequent consumption of milk, cottage cheese, white bread, rice, white pasta, cold meats, eggs, and fruit, while EPA children more often reached for vegetables, whole-grain bread, fish, but also sweets, fruit juices, and fast food. The mean frequency of consumption of food products, dishes, and beverages at the beginning of the study is provided in Supplementary Materials (Table S1).

Girls more often reported consuming vegetables (87.14% vs. 74.31%; *p* = 0.125), fruit (78.16% vs. 74.49%; *p* = 0.646), fish (64.41% vs. 52.89%; *p* = 0.271), milk (69.27% vs. 63.92%; *p* = 0.544), yogurt (86.79% vs. 83.25%; *p* = 0.614), and whole-grain bread (48.45% vs. 43.71%; *p* = 0.696). Boys more frequently chose meat and fatty foods. Poultry consumption was higher among boys (59.75% vs. 42.71%; *p* = 0.118), as was intake of cold cuts (69.35% vs. 61.68%; *p* = 0.414) and fried dishes (49.83% vs. 46.70%; *p* = 0.851). Boys also consumed eggs (61.99% vs. 60.51%; *p* = 1.000), fatty cheeses (66.14% vs. 61.83%; *p* = 0.673), and butter (94.58% vs. 88.08%; *p* = 0.220) more often than girls (see Supplementary Figure S1).

Boys showed higher intake of fruit juices (52.83% vs. 43.60%; *p* = 0.334) and sweetened beverages (32.25% vs. 30.78%; *p* = 0.861). Other products showed no notable differences: white bread (girls 92.02%; boys 93.07%; *p* = 1.000), red meat (40.75% vs. 39.10%; *p* = 0.851), cottage cheese (45.75% vs. 44.05%; *p* = 0.868), sweets (90.73% vs. 91.65%; *p* = 1.000), and fast food (36.11% vs. 32.34%; *p* = 0.700). Full results are presented in Supplementary Figure S1.

#### 3.3.2. Final

At the final measurement, children most frequently consumed four meals per day (EPA 49.98%; SPA 43.28%; *p* = 0.136). Three meals were reported by 28.12% of EPA and 27.89% of SPA children, while five meals were declared by 15.65% (EPA) and 28.83% (SPA). Breakfast consumption patterns were similar in both groups, with 72.0% of SPA and 65.44% of EPA children eating breakfast regularly (*p* = 0.061). About one-quarter consumed breakfast irregularly, and approximately 10% received money to purchase it.

Snacking habits were comparable. Fruit was the most common snack (EPA 41.86%; SPA 37.12%; *p* = 0.060), followed by snacks sweet (EPA 18.71%; SPA 20.60%; *p* = 0.089) and salty snacks (EPA 20.80%; SPA 18.56%; *p* = 0.096). Only about 10% did not snack (*p* = 0.140).

SPA children consumed milk more frequently than EPA children (40.18% vs. 27.90%; *p* = 0.005). Cottage cheese and yogurt consumption was higher in EPA and consumption of fatty cheeses was similar in both groups (EPA 19.50%; SPA 15.20%; *p* = 0.801). Daily butter consumption was very frequent in both groups (SPA 64.25%; EPA 57.90%; *p* = 0.734) (see Supplementary Table S2).

White bread intake was similar, while whole-grain bread was chosen more often by EPA children (16.28% vs. 11.31%; *p* = 0.030). EPA children more frequently consumed rice and small groats (*p* = 0.021) as well as whole-grain groats and oatmeal (*p* = 0.032) (see Supplementary Table S2).

Poultry dishes were more common in the EPA group (21.46%; SPA 17.60%; *p* = 0.022), while red meat and cold cuts consumption did not differ significantly. Fruit consumption was more frequent in SPA (57.63%) than EPA children (45.98%; *p* = 0.002), whereas vegetable intake was similar (see Supplementary Table S2).

Fruits and vegetables were a common part of the children’s daily diet. Fruit consumption was more frequent in SPA (57.63%) than EPA children (45.98%; *p* = 0.002), whereas vegetable intake was similar (see Supplementary Table S2).

SPA children ate fast food more often than EPA children (SPA 25.15%; EPA 20.10%; *p* = 0.038). Daily consumption of sweets remained high in both groups, but higher in SPA than EPA (SPA 58.0%; EPA 46.0%; *p* = 0.431). EPA children more frequently consumed sweetened beverages than SPA children (*p* = 0.273).

A higher proportion of SPA children reported healthy eating habits (82.44% vs. EPA 64.44%; *p* = 0.064), while poor nutritional behavior was more common in EPA (35.56%) than SPA children (17.56%; *p* = 0.024). Nearly all children believed that proper nutrition improves athletic performance.

On average, SPA children more often than EPA reached for milk and fruit at the end of the study, while EPA children more often chose whole-grain bread, groats, and poultry dishes. Significant differences between groups were found for the consumption of milk (*p* = 0.005), cottage cheese (*p* = 0.040), white bread (*p* = 0.012), whole-grain bread (*p* = 0.030), rice and small groats (*p* = 0.021), whole-grain groats and oatmeal (*p* = 0.032), poultry (*p* = 0.022), fruit (*p* = 0.002), and fast food (*p* = 0.038). No significant differences were found for the remaining products. The mean frequency of consumption of food products, dishes, and beverages at the end of the study is provided in Supplementary Materials (Table S2).

A comparison of nutritional behaviors between girls and boys showed largely similar patterns. Fruit consumption was comparable (girls 82.17%; boys 85.31%; *p* = 0.646), as was vegetable intake (86% vs. 87.75%; *p* = 0.125). White bread was consumed by most participants (girls 86.78%; boys 92.63%; *p* = 1.000), while whole-grain bread intake was also similar (44.18% vs. 38.83%; *p* = 0.696). Boys more often chose poultry dishes (67.30% vs. 52%; *p* = 0.118), whereas girls consumed red meat more frequently (49.18% vs. 44.65%; *p* = 0.025). Cold-cut consumption was slightly higher in girls (64.20% vs. 57.16%; *p* = 0.078). Fish intake was low in both sexes (girls 46.94%; boys 44.19%; *p* = 0.214) (see Supplementary Figure S2).

Consumption of fried meat and dough dishes was high in both groups (girls 44.38%; boys 47.25%; *p* = 0.314), and egg intake was similar (62.24% vs. 66.29%; *p* = 0.174). Girls more often consumed milk (77.55%; *p* = 0.410) and yogurt (89.95%; *p* = 0.470), while boys more frequently chose fatty cheeses (67.50%; *p* = 0.062) and butter (96.35%; *p* = 0.870). Sweets were consumed very frequently by both girls and boys (91.15% and 87.90%; *p* = 1.000). Fast-food intake was also comparable (25.22% vs. 28.37%; *p* = 0.700) (see Supplementary Figure S2).

Boys more frequently selected fruit juices (51.70% vs. 41.70%; *p* = 0.067) and sweetened, carbonated or not, beverages (33.10% vs. 28.30%; *p* = 0.971) than girls. Girls more often than boys chose vegetables, fruit, milk, yogurt, fish, as well as cold cuts and red meat dishes, while boys more often chose poultry, fried meat and dough dishes, fatty cheeses, carbonated drinks, and sweetened beverages. Both groups maintained a very high consumption of white bread and sweets (over 90%). Mean food and beverage consumption among girls and boys at the final of the study is provided in the Supplementary Materials (Figure S2).

#### 3.3.3. Changes in Nutritional Behavior

**Figure 3:**
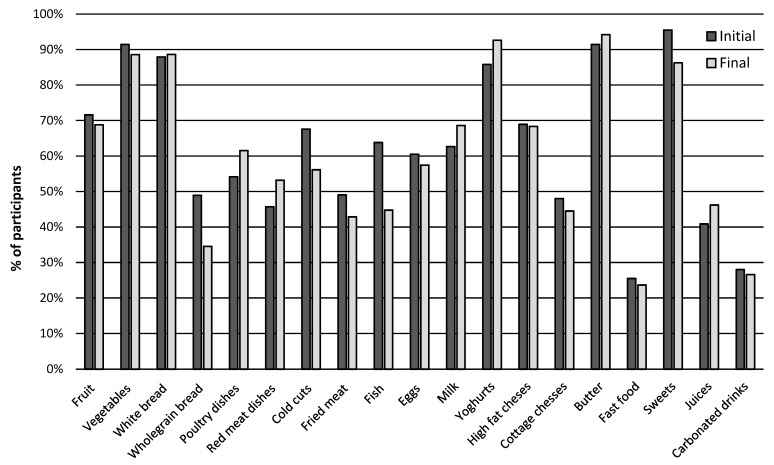
Food and beverage consumption at the beginning and end of the study in the EPA group. Frequently consumed products (milk, yogurt, full-fat cheeses, wheat bread, poultry, cold cuts, fried dishes, fruit, vegetables, butter, and sweets) are presented as the summed percentage of responses “several times a week” and “every day.” Products consumed less frequently (cottage cheese, whole-grain bread, eggs, fish, fast food, fruit juices, and carbonated drinks) are presented as a percentage of responses “once a week”.

Among children in the EPA group, white bread consumption remained high at both the beginning and the end of the study (87.91% and 88.61%, respectively). In contrast, a decrease was observed in the proportion of children consuming whole grain bread once a week (from 48.93% to 34.54%), accompanied by an increase in those consuming it several times a week (from 20.63% to 29.27%). There was also an upward trend in the consumption of poultry (from 54.15% to 61.54% and red meat (from 45.70% to 53.18%) (Figure 3).

At the same time, a decline was noted in the consumption of cold cuts (from 67.59% to 56.14%), fish (from 63.81% to 44.75%), and eggs (from 60.51% to 57.43%). The consumption of fried meat and dough-based dishes also decreased (from 49.05% to 42.85%).

Regarding dairy products, an increase was recorded in the consumption of milk (from 62.66% to 68.61%) and yogurt (from 85.80% to 92.60%), while the consumption of fatty cheeses remained stable (68.64%). Cottage cheese intake decreased slightly (from 48.00% to 44.50%), whereas butter consumption showed a minor increase (from 91.45% to 94.20%).

A decrease was also observed in the consumption of sweets (from 95.50% to 86.25%) and fast food (from 25.50% to 23.65%). Overall, the EPA children reported an increased consumption of dairy products (milk and yogurt) and certain types of meat (poultry and red meat), as well as a reduction in the intake of fish, cold cuts, and sweets.

**Figure 4:**
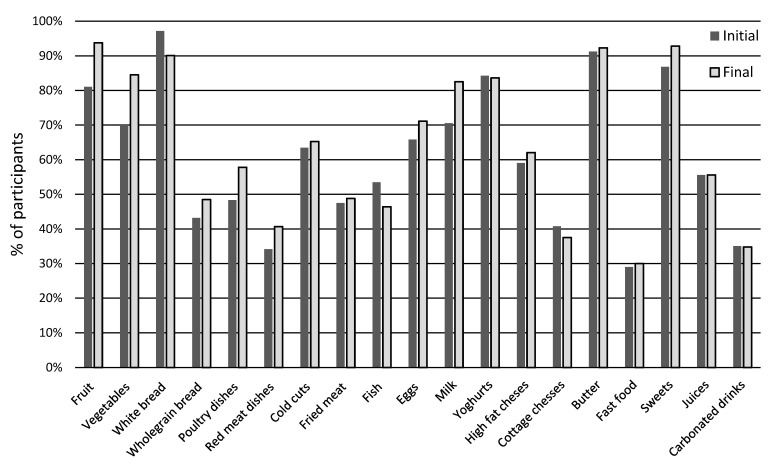
Food and beverage consumption at the beginning and end of the study in the SPA group. Frequently consumed products (milk, yogurt, full-fat cheese, wheat bread, poultry, cold cuts, fried dishes, fruit, vegetables, butter, and sweets) are presented as the summed percentage of responses “several times a week” and “daily.” Products consumed less frequently (cottage cheese, whole-grain bread, eggs, fish, fast food, fruit juices, carbonated drinks) are presented as a percentage of responses “once a week”.

In the SPA group, a significant increase was observed in the percentage of children regularly consuming fruit (from 81.04% to 93.77%) and vegetables (from 70.00% to 84.51%) (Figure 4). The frequency of whole-grain bread consumption slightly increased (from 43.19% to 48.47%), while white bread consumption decreased (from 97.17% to 90.08%).

In the case of fruit juices and sweetened drinks, the consumption remained unchanged (55.58% and 35%, respectively). There was an increase in the frequency of consumption of poultry dishes (from 48.31% to 57.80%), red meat dishes (from 34.15% to 40.65%), and eggs (from 65.80% to 71.11%). The consumption of cold cuts and fried dishes remained relatively stable (63.43% vs. 65.23% and 47.48% vs. 48.80%, respectively). However, a decrease in fish consumption was noted (from 53.49% to 46.39%) (Figure 3).

In the case of dairy products, an increase in milk consumption was observed (from 70.53% to 82.51%) and a slight increase in the consumption of fatty cheeses (from 59.05% to 62.04%). Yogurt consumption remained stable (84.24% vs. 83.60%), while a decrease was noted for cottage cheese (from 41.00% to 37.50%). The frequency of butter consumption remained unchanged (91.23% vs. 92.30%).

Unfavorable trends were observed in the frequency of sweets consumption (from 86.85% to 92.80%) and fast food consumption (from 28.99% to 30%). In summary, an increase in the consumption of fruits, vegetables, milk, and whole-grain bread was observed among children in the SPA group, while a simultaneous decrease in the consumption of wheat bread and fish was observed. On the other hand, there was an increase in the percentage of children eating sweets and fast food.

Comparison of both groups reveals certain similarities—both groups observed an increase in the consumption of milk and meat (poultry and red meat), as well as a decrease in the consumption of fish and cottage cheese. However, there were different trends in the frequency of fruit, vegetables, and sweets consumption in both groups. The EPA children consumed these products less frequently, while the SPA children consumed them more frequently. The results indicate that the SPA children exhibited a greater improvement in their nutritional behavior regarding fruit and vegetables, whereas the EPA group exhibited a tendency to restrict sweets, albeit at the expense of a decreased consumption of plant foods and fish.

### 3.4. PCA

**Figure 5:**
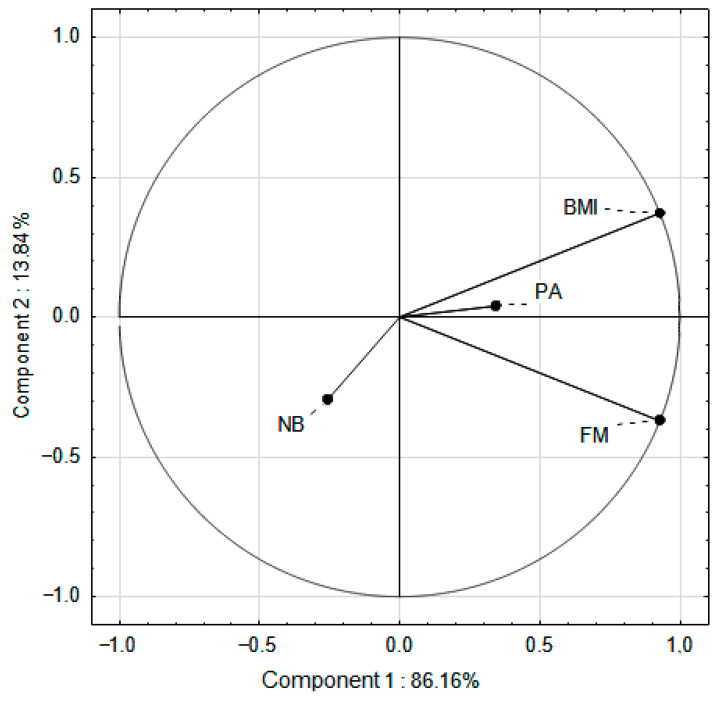
PCA of BMI, FM, PA, favorable nutritional behavior in relation to standard PA in school hours (SPA group). PA—physical activity; BMI—Body Mass Index; FM—Fat Mass; NB—favorable nutritional behavior.

In the PCA, vectors located on opposite sides of the coordinate system indicated a negative correlation. In the SPA group, the strongest association was observed between BMI and FM (Figure 5), suggesting that an increase in BMI was significantly correlated with an increase in FM. Physical activity (PA) was positioned in the same half-plane as BMI and FM, indicating that lower PA levels were associated with higher BMI and FM values. In contrast, the vector representing favorable nutritional behaviors (NB) was oriented in the opposite direction to BMI and FM. The results did not allow us to clearly confirm the relationship, although it was observed that better eating behaviors were associated with lower BMI and FM values.

**Figure 6:**
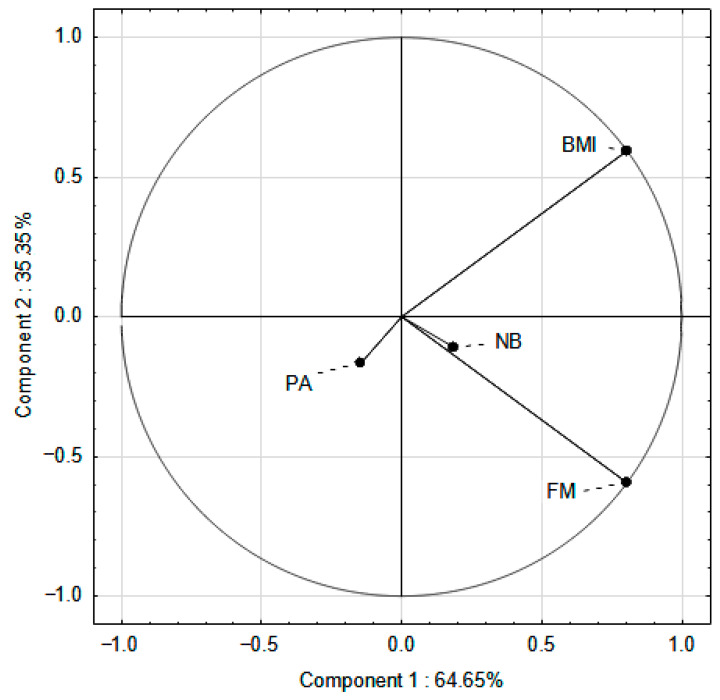
PCA of BMI, FM, PA, favorable nutritional behavior in relation to increased PA in school hours (EPA group). PA—physical activity; BMI—Body Mass Index; FM—Fat Mass; NB—favorable nutritional behavior.

In the EPA group, the strongest association was observed between BMI and FM, indicating that an increase in BMI was accompanied by an increase in FM (Figure 6). The NB vector was oriented in the opposite direction to FM, suggesting that favorable nutritional behaviors were associated with a decrease in FM. A weak correlation was found between NB and PA, indicating that a higher level of PA was only slightly associated with an improvement in nutritional behaviors.

## 4. Discussion

The findings of this study showed that among overweight and obese children aged 11 years, increased PA during school hours significantly influenced the direction of changes in body weight and nutritional behaviors. On average, after 12 months of intervention, children in the intervention group demonstrated stable body weight and reduced FM, while those in the control group experienced a systematic increase in body weight and FM. These disparities were also evident in BMI levels. The intervention group exhibited a trend toward a decrease in BMI in comparison to the baseline, whereas the control group experienced an additional increase. Our findings are in agreement with prior research that has demonstrated the efficacy of school-based interventions in fostering PA and fitness among obese children [46].

### 4.1. The Impact of Increased PA on BMI and FM

After 12 months of intervention, children in the increased physical activity (EPA) group maintained stable BMI and lower FM levels, while those in the control group (SPA) increased their BMI. The decreased percentage of obese children and the increased percentage of overweight children in the EPA group may indicate a positive effect of moderate physical activity on improving body composition. These results are consistent with previous reports confirming the beneficial effects of school-based interventions on children’s body weight and physical fitness [47,48,49,50]. At the same time, they confirm the observations of Janssen and LeBlanc [51] that the relationship between physical activity and obesity reduction may be moderate and require longer follow-up.

Additional investigations and observations regarding obese children who are subjected to PA intervention are required.

### 4.2. The Impact of Increased PA at School on Reducing Body Fat

The observed changes in body fat confirm the effectiveness of increased physical activity in reducing body fat accumulation. The EPA group experienced an average decrease in body fat of 1%, while the SPA group experienced a 1% increase. This effect was particularly pronounced in boys in the EPA group. Similar results were obtained by Rutkowski et al. (2019) [52] Méndez-Hernández (2022) [53] and Soares et al. (2023) [54], confirming the beneficial effect of physical activity programs on body composition.

The differences between our results and other studies (e.g., Wang et al. 2022 [47]) may be due to the age of the participants, the duration of the intervention, and the lack of a parallel nutritional education component that could further enhance the effects. Some studies suggest that a significant increase in energy expenditure in children with higher BMI effectively reduces FM, which directly leads to a reduction in body fat [55].

### 4.3. The Impact of Increased PA at School on Nutritional Behawior

It is widely acknowledged that unhealthy nutritional behavior combined with low physical activity (PA) is strongly associated with the development of obesity [12,13,14,15]. Our findings align with previous research indicating that PA may promote healthier food choices, such as increased consumption of vegetables and whole grains [15,16]. However, the observed improvements were only partial and did not eliminate established unhealthy habits, particularly the excessive intake of sweets and sugar-sweetened beverages [56,57].

This limited effect may be explained by several factors. First, nutritional behavior is shaped not only by school-based interventions but also by family habits and the home food environment, which were not modified during the study [30,46,48,58]. Second, children’s food preferences tend to be resistant to short-term behavioral change, especially in the absence of targeted nutritional education [30,46,48,58]. Third, socio-economic and environmental factors—such as food availability, parental modeling, and peer influence—likely moderated the impact of the intervention [30,46,48,58].

The higher consumption of energy-dense foods such as sweets, red meat, and sweetened beverages in the control group confirms that less physically active children tend to maintain less favorable dietary patterns [58,59]. Similar effects were also described in studies where participation in sports programs was associated with higher consumption of yogurt and milk [60]. Nevertheless, while the intervention group demonstrated certain positive trends (increased intake of fruit, whole grains, and dairy products; reduced intake of sweets and fast food), the absence of a structured dietary education component may explain why these changes were modest and inconsistent across food categories.

These findings are consistent with the meta-analysis by Mead et al. (2017), which concluded that exercise-based interventions without concurrent nutritional education have limited effects on improving diet quality [61,62]. Therefore, future programs should integrate physical activity with parental involvement and nutritional education to achieve more sustainable behavioral change.

In the control group, an unfavorable trend was observed, characterized by an increase in the daily intake of sweets and red meat. This finding is consistent with previous studies indicating that school-aged children tend to prefer high-calorie snacks and red meat, which may reflect their growing nutritional autonomy and exposure to peer and media influences [63,64]. In the intervention group, a modest improvement was found, including a higher intake of fermented dairy products, poultry, and whole-grain foods, accompanied by a decrease in sweets, juices, sweetened beverages, and fast-food consumption. However, frequent consumption of fried meats, pastries, full-fat cheeses, and salty snacks persisted.

These results suggest that although the school-based increase in physical activity (PA) had a beneficial impact on body composition, its effect on dietary behaviors was limited. One possible explanation is that physical activity alone, without simultaneous nutritional education or family engagement, is insufficient to drive meaningful dietary change [30,58]. Children’s eating habits are shaped by multiple determinants—including parental modeling, household food availability, and socio-economic status—that were not modified during this intervention [30,58]. Moreover, dietary behavior tends to be resistant to short-term change and often requires targeted, repeated educational reinforcement [24,61].

Our findings align with the meta-analysis by Mead et al. (2017), which showed that exercise-only interventions produce minimal effects on BMI and fat mass reduction compared to multifaceted programs combining PA with dietary education [61]. Similar conclusions were reached by O’Brien et al. (2021), emphasizing the need for integrated school-based interventions that address both nutrition and physical activity simultaneously [62].

While the intervention in our study led to a measurable reduction in the number of obese children, the observed improvements in nutritional behavior were modest. This indicates that improved physical fitness and weight reduction do not necessarily translate into healthier dietary choices. Future interventions should therefore incorporate parental involvement, nutritional education, and strategies addressing the broader social and environmental determinants of health to achieve more sustainable behavioral change.

The results obtained in our study indicated that an intervention based solely on increasing PA in the school setting produced measurable benefits in terms of weight and body fat reduction, but its impact on nutritional behavior was limited. The lack of significant changes in this area may be due to the children’s established eating patterns and the lack of a direct nutritional education component in the implemented program. Future research should consider multi-component interventions combining PA, nutritional education, and support from the family and school environment, which may more effectively support lasting lifestyle changes.

Our findings extend previous research on school-based interventions by demonstrating that increased physical activity during school hours may indirectly influence children’s nutritional behavior. This relationship has been observed in some earlier studies, although the mechanisms and magnitude of the effect remain inconsistent across research designs [65].

Despite the abundance of observations that our investigation produced, it was not without its limitations. First, the study was limited to a relatively small sample of participants. The investigation encompassed only children between the ages of 10.90 and 11.90. The study could have been expanded to include adolescents of varying ages and a broader geographical area. One PE lesson could not accommodate all of them for classes of 21–24 kids.

We did not evaluate the attendance at PE or other lessons, nor did not include extracurricular PA. Objective monitoring of attendance in physical education classes and extracurricular physical activity was not included in this part of the project; these data, together with detailed fitness test results, constitute a separate component of the study and will be reported in an upcoming publication.

Gender was not balanced between groups, and participants were not stratified by gender. Medical examinations were not performed, and maturation status (pubertal stage) was not assessed, which may be relevant at the age of 11. Information on previous sports participation, extracurricular training, and psychological or behavioral difficulties was not collected. These factors may influence both dietary behaviors and engagement in physical activity and should be addressed in future research.

The authors are aware that prior to conducting the main study, validation of the questionnaire used was necessary. Collecting quantitative data from children of this age is particularly challenging due to their limited ability to accurately assess portion sizes and frequent memory distortions. The lack of validation of the tool in the studied age group could have led to bias and limited the comparability of results with other studies. Furthermore, self-assessment of dietary behaviors is subject to social desirability bias—children may overestimate healthy dietary choices or underestimate the frequency of consuming inappropriate foods, such as sweets or sweetened beverages. It is worth noting, however, that all questionnaires were completed face-to-face in the presence of a trained interviewer, who provided explanations and ensured that all questions were understood correctly. This procedure helped minimize potential misunderstandings and improved the reliability of the responses, despite the self-reported and anonymous nature of the tool.

Regrettably, it is exceedingly challenging to accumulate quantitative data from children. Their own subjective statements were the sole source of information regarding children’s nutritional behavior. Future studies should consider using validated, standardized nutritional tools adapted to children’s age, as well as individual codes that allow for anonymous but consistent tracking of changes in nutritional behavior and somatic parameters over time. This approach would increase the reliability and validity of conclusions regarding the relationship between physical activity, diet, and nutritional status.

The nutritional behavior assessment questionnaire used, based on self-assessment and frequency of consumption, did not provide quantitative data and was not validated in the study population, which might increase the risk of bias. Another limitation is the possibility of social desirability bias, which involves potential overreporting of healthy nutritional behaviors during children’s self-assessment. The anonymity of the survey was also a significant limitation. While this allowed for increased honesty in responses, it also prevented individual correlation of questionnaire results with BMI and body fat percentage. Consequently, the analysis was based solely on group mean values, without the ability to assess the relationship between changes in nutritional behavior and individual changes in body composition. This limited the ability to fully understand the mechanisms by which increased physical activity affects nutritional behavior and the potential interactions between these variables. The research should include an analysis of the relationship between nutritional behavior and the nutritional status of children. However, the nutritional status was not examined. It is frequently believed that the nutritional behavior of children and their families may be influenced by their economic circumstances; however, this study did not investigate this question.

The limited impact of the intervention on children’s nutritional behaviors may be due to several external factors that were not directly controlled in this study. Particular attention should be paid to the role of family and the home environment, which largely shape children’s eating habits. Even increasing physical activity at school may have limited impact if daily food choices at home remain unfavorable.

Future studies should consider using validated, standardized nutritional tools adapted to children’s age, as well as individual codes that allow for anonymous but consistent tracking of changes in nutritional behavior and somatic parameters over time. This approach would increase the reliability and validity of conclusions regarding the relationship between physical activity, diet, and nutritional status.

The school environment may also play a significant role—the availability of convenience stores, vending machines, and school cafeteria meals can contribute to the perpetuation of less healthy patterns. The socioeconomic status of families is also important, influencing both the ability to purchase health-promoting products and parents’ nutritional awareness.

A significant limitation of our study was the PPS protocol used, which included only data from participants who strictly adhered to the study protocol. The PPS method allowed us to assess the direct effect of the intervention under optimal conditions, which was among those who consistently participated in the program. Nevertheless, the generalizability of the findings might be restricted by this analytical choice, as the results primarily reflect the outcomes of compliant participants rather than the entire target population of overweight and obese children. This approach was selected due to the considerable amount of missing data among non-completers, which made the application of an Intention-to-Treat (ITT) analysis impractical.

The ITT approach was not applied just because a considerable number of participants did not complete all required assessments, leading to substantial missing data. Applying ITT under these conditions could reduce the reliability of the results. Future studies should be designed using an ITT protocol, which includes all participants randomly assigned to groups, regardless of their adherence to the study protocol.

The authors realize that using a 24 h recall would be a more accurate method of assessing nutritional behavior. Further research should be based on this method and should concentrate on the long-term monitoring of overweight children, their nutritional behavior, and their body composition.

Another limitation concerns the representativeness of the study sample. The participants were recruited from a limited geographic region and a narrow age range, which may restrict the generalizability of the results to other populations of overweight and obese children.

## 5. Conclusions

The present study suggests that participation in an extended school-based physical activity (PA) program may be associated with more favorable changes in BMI and fat mass (FM) categories among children who were overweight and obese. A higher proportion of children in the intervention group showed a shift from obesity to overweight compared with the control group. However, improvements in nutritional behavior were modest and should be interpreted with caution, as they were based on a self-reported, partially validated questionnaire.

Although the extended physical activity during school hours was correlated with a decrease in the number of obese children, the observational nature of the study precludes the drawing of definitive conclusions. The intervention may have contributed to an increase in energy expenditure, which could account for the improvements in BMI and FM. Nevertheless, the impact on nutritional behavior was limited.

Overall, these findings indicate that higher amounts of school-based PA may be linked with measurable improvements in body composition but only minor changes in dietary habits in this population. Given the study’s design, sample characteristics, and methodological constraints, the results cannot be generalized beyond the specific group studied.

Future research should combine extended PA interventions with structured nutritional education and behavioral support. Long-term, multi-center studies using fully validated dietary assessment tools and intention-to-treat (ITT) approaches are needed to strengthen the evidence base and evaluate the sustainability of potential effects.

## Figures and Tables

**Table 1:** Comparison of characteristics of participants who completed the study and those who dropped out (dropout analysis).

Indicator	SPA			EPA		
	Completed (*n* = 69)	Dropped Out (*n* = 88)	*p*	Completed (*n* = 43)	Dropped Out (*n* = 60)	*p*
Height (cm)	155.95	156.26	0.78	153.15	153.06	0.940
Weight (kg)	61.59	62.37	0.620	54.11	54.08	0.987
FM (kg)	19.13	19.61	0.470	14.94	14.72	0.800
Fat Mass (%)	31.03	31.44	0.651	27.53	27.21	0.681
BMI (kg/m^2^)	25.08	25.63	0.401	23.07	23.11	0.910

EPA—elevated PA group; SPA—standard PA group; BMI—body mass index; FM—fat mass; *p*—*t*-test; *p* ≤ 0.05.

**Table 2:** BMI categories in the baseline and the final measurement sessions.

Measurement Session	SPA			EPA			*p* *
	Girls	Boys	Mean	Girls	Boys	Mean	
Overweight							
Initial	58.95%	57.50%	58.22%	59.25%	55.20%	57.22%	0.170
Final	53.50%	58.25%	55.87%	61.50%	62.80%	62.15%	0.071
Obesity							
Initial	41.05%	42.50%	41.77%	40.75%	48.80%	44.77%	0.245
Final	46.50%	41.75%	44.13%	38.50%	37.24%	37.87%	0.050
A change in the BMI category from overweight to normal							
Entire study period	0	0	0	3.70%	12.5%	8.10%	0.000

EPA—elevated PA group; SPA—standard PA group; *n*—number of participants; * *p*—*t*-test; *p* ≤ 0.05.

**Table 3:** Anthropometric indicators of children in the baseline and final measurements.

Indicator	Baseline All Group (95% CI)	Final All Group (95% CI)	*p*	SPA			EPA		
				Baseline (95% CI)	Final (95% CI)	*p*	Baseline (95% CI)	Final (95% CI)	*p*
Height (cm)	151.42 (4.75–8.31)	157.65 (7.68–9.25)	0.061	152.50 (6.29–8.18)	159.41 (7.02–9.02)	0.077	150.42 (5.03–8.52)	155.88 (5.66–7.67)	0.067
Weight (kg)	54.38 (7.63–9.29)	61.32 (9.25–10.15)	0.042	57.81 (5.61–9.47)	65.37 (8.12–11.18)	0.034	50.94 (3.85–5.16)	57.28 (5.33–9.25)	0.060
FM (kg)	29.35 (4.00–5.31)	29.21 (6.25–9.02)	0.871	30.62 (2.32–3.19)	31.44 (6.12–7.18)	0.069	28.08 (4.19–5.85)	26.98 (4.25–8.25)	0.074
Fat Mass (%)	16.01 (3.31–4.32)	18.07 (7.54–9.45)	0.074	17.67 (2.77–4.68)	20.59 (7.66–9.14)	0.057	14.35 (2.81–5.75)	15.54 (3.35–6.69)	0.087
BMI (kg/m^2^)	23.58 (1.09–2.90)	24.57 (2.40–3.19)	0.690	24.65 (1.34–2.26)	25.51 (3.74–6.25)	0.097	22.51 (1.26–2.59)	23.62 (1.70–3.47)	0.098
BMI (percentile)	90th (2.14–3.80)	97th (2.35–3.14)	0.078	97th (3.18–4.36)	97th (2.55–3.17)	0.740	90th (2.97–3.14)	90th (1.58–2.66)	0.840

EPA—elevated PA group; SPA—standard PA group; *n*—number of participants; BMI—body mass index; FM—fat mass; 95% CI—confidence interval; *p* ≤ 0.05.

## Data Availability

The original contributions presented in this study are included in the article and Supplementary Materials. Further inquiries can be directed to the corresponding author.

## Supplementary Materials

The following supporting information can be downloaded at: https://www.mdpi.com/article/10.3390/nu17243905/s1, Table S1: Average frequency of consumption of food products, dishes and beverages in EPA and SPA at the baseline of the study, %; Table S2: An average frequency of consumption of food products, dishes and beverages in EPA and SPA at the end of the study, %; Figure S1: Average consumption of products and beverages among girls and boys at the baseline of the study; Figure S2: Average food and beverage consumption among girls and boys at the final of the study.

## Abbreviations

The following abbreviations are used in this manuscript:

BMI	Body Mass Index
EPA	Elevated Physical Activity group
FM	Fat Mass
PA	Physical Activity
PCA	Principal Component Analysis
PE	Physical Education
PF	Physical Fitness
SPA	Standard Physical Activity group
