# Supplementary material for: The Impact of Physical Activity at School on the Nutritional Behavior of Overweight Children
Source: Nutrients. 2025 Dec 13;17(24):3905. doi: 10.3390/nu17243905 (PMC12736377; doi:10.3390/nu17243905)
Supplement: Supplementary file 1 [file nutrients-17-03905-s001.zip › nutrients-4035082-supplementary.pdf]

Table S1. Average frequency of consumption of food products, dishes and beverages in EPA and SPA at the baseline of the study, %.

No	Foods	**Group**	Frequency of consuming food products [%]					Statistical measures*
			Never	1–3 times a month	Once a week	Several times a week	Every day	
1.	Milk	SPA	3.87	0.00	25.60	38.83	31.70	χ^2= 14.802 df-4, p=0.075, VC=0.177
		EPA	3.69	13.47	20.17	31.95	30.71	
2.	Yoghurts (natural and flavoured)	SPA	0.00	2.81	13.45	52.70	31.54	χ^2= 0.776 df-4, p=0.941 VC=0.041
		EPA	0.00	2.80	11.40	48.70	37.10	
3.	Cottage chesses	SPA	4.75	5.00	40.75	33.65	15.64	χ^2= 3.055 df-4, p=0.548 VC=0.082
		EPA	2.25	8.75	46.00	26.70	16.50	
4.	High fat chesses (including hard, processed and blue chesses)	SPA	0.00	10.58	30.39	45.52	13.63	χ^2= 3.268 df-4, p=0.031 VC=0.085
		EPA	0.00	6.48	24.60	47.80	21.13	
5.	White bread and rolls	SPA	0.00	0.00	2.83	25.87	71.30	χ^2= 7.606 df-4, p=0.107 VC=0.130
		EPA	0.00	0.00	12.08	16.93	70.98	
6.	Whole grain bread and rolls	SPA	19.72	20.75	43.19	9.59	6.75	χ^2= 15.709 df-4, p=0.034 VC= 0.187
		EPA	7.96	9.47	48.97	20.63	13.24	
7.	White rice, white pasta, fine-grained groats	SPA	0.93	1.85	45.70	37.25	14.27	χ^2= 24.154 df-4, p=0.001 VC=0.232
		EPA	2.54	7.39	57.12	22.17	10.78	
8.	Coarse-grained groats, buckwheat groats, oatmeal	SPA	7.57	9.59	56.86	18.19	7.79	χ^2= 6.811 df-4, p=0.014 VC= 0.123
		EPA	1.85	6.08	61.20	27.17	3.69	
9.	Poultry meat dishes	SPA	0.00	13.56	38.13	34.15	14.16	χ^2= 1.735 df-4, p=0.084 VC=0.062
		EPA	0.00	12.08	28.02	36.75	17.40	
10.	Red meat dishes	SPA	6.60	13.15	34.15	27.15	18.95	χ^2= 2.325 df-4, p=0.676 VC=0.072
		EPA	2.60	5.55	45.70	28.00	18.15	
11.	Cold cuts, sausages, frankfurters	SPA	0.00	6.10	30.48	28.72	34.71	χ^2= 3.596 df-4, p=0.963 VC = 0.036
		EPA	0.00	15.93	16.47	34.10	33.49	
12	Friedmeat and dough dishes	SPA	0.00	17.45	35.08	44.85	2.63	χ^2= 6.766 df-4, p=0.942 VC=0.041
		EPA	0.00	14.16	36.35	44.75	4.30	
13.	Eggs	SPA	0.00	1.91	65.80	30.45	1.85	χ^2= 15.967 df-4, p=0.071 VC=0.188
		EPA	6.00	11.24	60.51	22.25	0.00	
14.	Fish	SPA	25.76	17.92	53.49	2.83	0.00	χ^2= 12.513 df-4, p=0.063 VC=0.167
		EPA	9.39	17.86	63.81	6.24	2.69	
15.	Legumes	SPA	53.50	44.25	2.25	0.00	0.00	χ^2= 0.766 df-4, p=0.942 VC = 0.041
		EPA	47.00	49.75	3.25	0.00	0.00	
16.	Fruit	SPA	0.00	0.98	17.97	38.94	42.10	χ^2= 9.682 df-4, p=0.546 VC=0.147
		EPA	0.00	11.63	16.78	32.95	38.64	
17.	Vegetables	SPA	5.66	0.00	24.35	30.56	39.43	χ^2= 13.302 df-4, p=0.009 VC=0.172
		EPA	0.00	0.00	8.54	39.88	51.58	
18.	Butter as an addition to bread, dishes, for frying, baking, etc.	SPA	0.00	3.50	5.28	31.50	59.73	χ^2= 0.191 df-4, p=0.995 VC = 0.020
		EPA	0.00	4.25	4.25	31.00	60.50	

19.	Lard as an addition to bread, dishes, for frying, baking, etc.	SPA	84.83	15.23	0.00	0.00	0.00	χ^2= 0.075 df-4, p=1.230 VC=0.021
		EPA	84.80	15.20	0.00	0.00	0.00	
20.	Fast food, different types	SPA	3.87	45.34	28.99	21.80	0.00	χ^2= 14.984 df-4, p=0.079 VC=0.182
		EPA	4.54	50.46	25.50	19.50	0.00	
21.	Sweets, candies, chocolate, bars	SPA	0.00	0.00	13.15	36.45	50.40	χ^2= 4.656 df-4, p=0.324 VC=0.101
		EPA	0.00	0.00	4.50	40.65	54.85	
22.	Juices	SPA	6.35	11.62	55.58	18.95	7.50	χ^2= 13.664 df-4, p=0.028 VC=0.174
		EPA	0.00	14.15	40.85	32.35	12.65	
23.	Sweetened carbonated or non-carbonated drinks	SPA	16.50	21.50	35.03	16.86	10.13	χ^2= 10.764 df-4, p=0.131 VC=0.103
		EPA	12.30	35.00	28.00	14.75	9.95	

*Chi squared test (χ^2); p-value ≤ 0.05; df — degrees of freedom; VC — V Cramera relationship strength

Table S2. An average frequency of consumption of food products, dishes and beverages in EPA and SPA at the end of the study, %.

No	Foods	Group	Frequency of consuming food products [%]					Statistical measures*
			Never	1–3 times a month	Once a week	Several times a week	Every day	
1.	Milk	SPA	4.10	0.00	13.39	42.33	40.18	χ^2= 14.810 df-4, p=0.005 VC=0.003
		EPA	0.00	3.19	28.20	40.71	27.90	
2.	Yoghurts (natural and flavoured)	SPA	0.00	6.40	10.00	50.25	33.35	χ^2= 7.637 df-4, p=0.105 VC=0.130
		EPA	0.00	0.00	7.40	51.35	41.25	
3.	Cottage chesses	SPA	4.75	6.50	37.50	34.45	17.00	χ^2= 11.032 df-4, p=0.040 VC=0.094
		EPA	1.00	5.26	44.50	28.75	20.60	
4.	High fat chesses (including hard, processed and blue chesses)	SPA	0.00	11.98	26.00	46.84	15.20	χ^2= 1.638 df-4, p=0.801 VC=0.060
		EPA	0.00	7.58	24.08	48.85	19.50	
5.	White bread and rolls	SPA	2.04	2.04	5.10	23.65	67.16	χ^2= 14.333 df-4, p=0.012 VC=0.098
		EPA	0.00	0.00	11.39	20.33	68.28	
6.	Whole grain bread and rolls	SPA	7.25	9.29	48.47	23.65	11.31	χ^2= 14.809 df-4, p=0.030 VC= 0.103
		EPA	13.66	6.25	34.54	29.27	16.28	
7.	White rice, white pasta, fine-grained groats	SPA	0.00	1.02	49.63	31.86	17.49	χ^2= 12.070 df-4, p=0.021 VC=0.164
		EPA	2.13	4.21	56.14	27.10	10.42	
8.	Coarse-grained groats, buckwheat groats, oatmeal	SPA	4.15	7.19	58.91	25.64	4.12	χ^2= 14.683 df-4, p=0.032 VC= 0.102
		EPA	7.27	5.19	57.38	19.74	10.42	
9.	Poultry meat dishes	SPA	0.00	0.00	42.20	40.24	17.56	χ^2= 11.365 df-4, p=0.022 VC=0.159
		EPA	0.00	5.23	28.05	40.08	21.46	
10.	Red meat dishes	SPA	0.00	6.35	40.65	30.80	22.20	χ^2= 8.691 df-4, p=0.069 VC=0.139
		EPA	0.00	6.12	53.18	25.60	15.10	
11.	Cold cuts, sausages, frankfurters	SPA	0.00	0.00	34.78	34.40	30.83	χ^2= 20.312 df-4, p=0.091 VC = 0.212
		EPA	9.31	8.29	26.25	29.98	26.16	
12	Friedmeat and dough dishes	SPA	0.00	13.78	37.43	45.10	3.70	χ^2= 2.97 df-4, p=0.562 VC=0.081
		EPA	0.00	20.68	36.50	41.75	1.07	
13.	Eggs	SPA	1.02	8.29	71.11	18.56	1.02	χ^2= 6.789

		EPA	4.08	10.42	57.43	22.88	5.19	df-4, p=0.147 VC=0.123
14.	Fish	SPA	20.62	26.81	46.39	5.17	1.02	χ^2= 8.218 df-4, p=0.083 VC=0.135
		EPA	17.74	37.52	44.75	0.00	0.00	
15.	Legumes	SPA	54.25	42.65	3.10	0.00	0.00	χ^2= 4.468 df-4, p=0.346 VC = 0.099
		EPA	41.07	51.43	7.50	0.00	0.00	
16.	Fruit	SPA	1.02	0.00	5.21	36.14	57.63	χ^2= 21.519 df-4, p=0.002 VC=0.219
		EPA	2.04	2.04	27.10	22.84	45.98	
17.	Vegetables	SPA	4.15	0.00	11.35	41.33	43.18	χ^2= 4.300 df-4, p=0.366 VC=0.098
		EPA	0.00	0.00	11.44	41.73	46.83	
18.	Butter as an addition to bread, dishes, for frying, baking, etc.	SPA	0.00	4.25	3.45	28.05	64.25	χ^2= 2.004 df-4, p=0.734 VC = 0.066
		EPA	0.00	2.25	3.55	36.30	57.90	
19.	Lard as an addition to bread, dishes, for frying, baking, etc.	SPA	88.02	11.98	0.00	0.00	0.00	χ^2= 0.026 df-4, p=0.991 VC=0.007
		EPA	88.75	11.25	0.00	0.00	0.00	
20.	Fast food, different types	SPA	2.04	42.88	29.93	25.15	0.00	χ^2= 10.142 df-4, p=0.038 VC=0.150
		EPA	11.96	44.29	23.65	20.10	0.00	
21.	Sweets, candies, chocolate, bars	SPA	0.00	0.00	7.20	34.80	58.00	χ^2= 3.817 df-4, p=0.431 VC=0.092
		EPA	0.00	0.00	13.75	40.25	46.00	
22.	Juices	SPA	7.13	11.60	47.23	21.70	7.35	χ^2= 9.010 df-4, p=0.060 VC=0.141
		EPA	0.00	16.55	46.18	29.08	8.23	
23.	Sweetened carbonated or non-carbonated drinks	SPA	18.25	20.00	34.80	14.10	12.85	χ^2= 5.136 df-4, p=0.273 VC=0.107
		EPA	18.70	33.15	26.60	12.60	8.95	

*Chi squared test (χ^2); p-value ≤ 0.05; df — degrees of freedom; VC — V Cramera relationship strength.

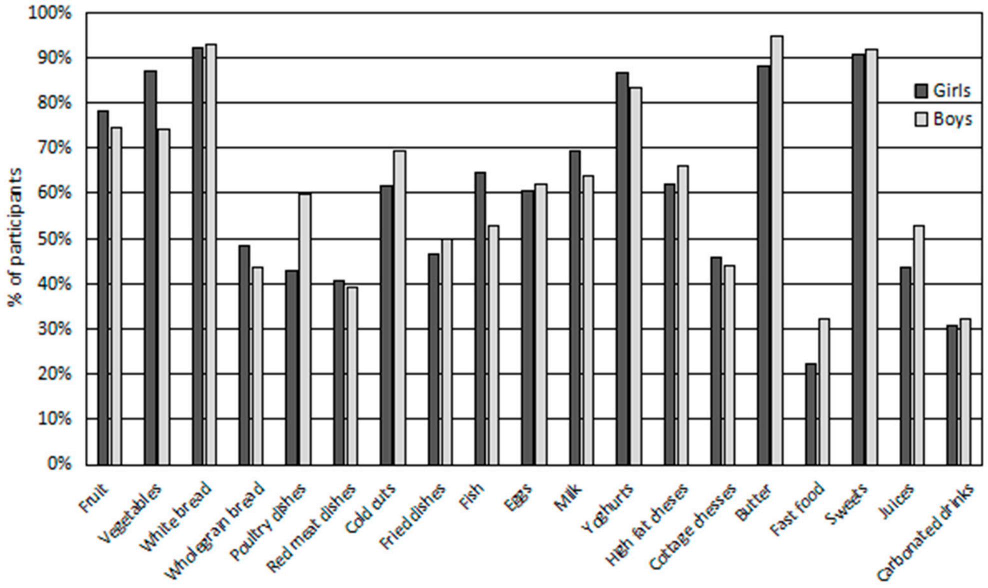

Figure S1. Average consumption of products and beverages among girls and boys at the baseline of the study. Frequently consumed products (milk, yogurt, full-fat cheeses, wheat bread, poultry, cold cuts, fried dishes, fruit, vegetables, butter, sweets) are presented as a summed percentage of responses "several times a week" and "every day." Products consumed less frequently (cottage cheese, whole-grain bread, eggs, fish, fast food, fruit juices, carbonated drinks) are presented as a percentage of responses "once a week.".

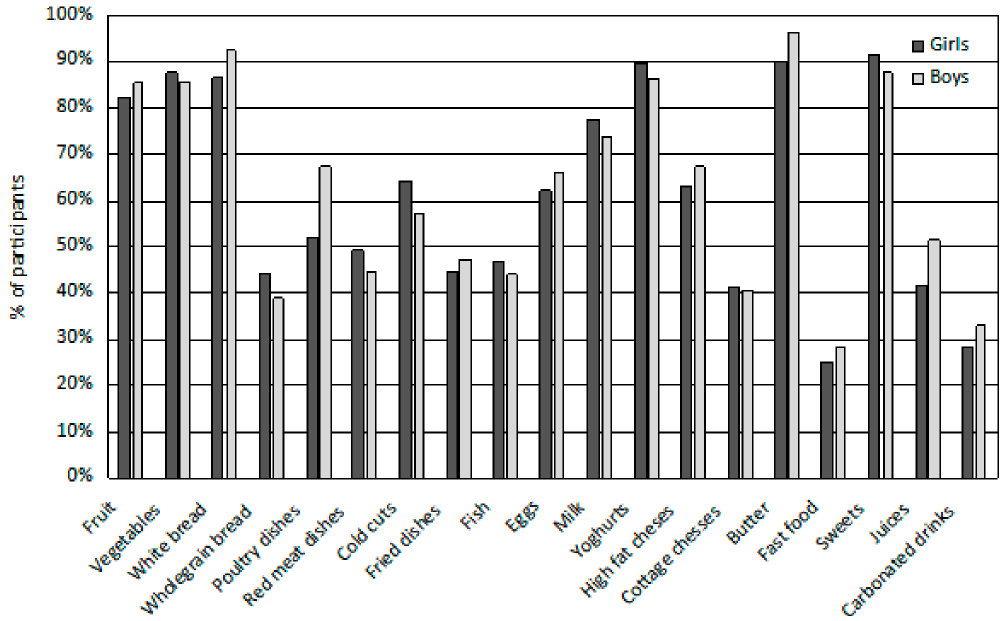

Figure S2. Average food and beverage consumption among girls and boys at the final of the study. Frequently consumed products (milk, yogurt, full-fat cheeses, wheat bread, poultry, cold cuts, fried dishes, fruit, vegetables, butter, sweets) are presented as summed percentage of responses "several times a week" and "every day." Products consumed less frequently (cottage cheese, whole-grain bread, eggs, fish, fast food, fruit juices, carbonated drinks) are presented as a percentage of responses "once a week.".
